# The Effects of Single Strains and Mixtures of Probiotic Bacteria on Immune Profile in Liver, Spleen, and Peripheral Blood

**DOI:** 10.3389/fnut.2022.773298

**Published:** 2022-04-12

**Authors:** Fiona Long Yan Fong, Hani El-Nezami, Otto Mykkänen, Pirkka V. Kirjavainen

**Affiliations:** ^1^School of Biological Sciences, The University of Hong Kong, Pokfulam, Hong Kong, SAR China; ^2^Institute of Public Health and Clinical Nutrition, University of Eastern Finland, Kuopio, Finland; ^3^Department of Environmental Health, Finnish Institute for Health and Welfare, Kuopio, Finland

**Keywords:** probiotic bacteria, immunomodulation, functional foods, *Lactobacillus rhamnosus*, *Bifidobacterium breve*, *Propionibacterium freudenreichii* ssp. *shermanii* JS, *Escherichia coli* Nissle 1917

## Abstract

Probiotic bacteria have potential use as immunomodulators but comparative data on their immunological effects are very limited. The aim of this study was to characterize the effect of oral administration of probiotic strains, alone or as mixtures, on systemic and organ-specific immune responses. For this purpose, healthy C57BL/6 mice were perorally administered probiotics for 3 weeks. A total of five common probiotic strains, *Lactobacillus rhamnosus* species GG (LGG) and LC705, *Bifidobacterium breve* 99 (Bb99), *Propionibacterium freudenreichii Shermanii* JS (PJS), and *Escherichia coli* Nissle 1917 (EcN), and two of their mixtures, were tested. Livers, spleens, and blood were collected for investigation. A number of five treatments increased the abundance of the natural killer (NK) cells. Bb99 had the most prominent effect on hepatic NK cells (20.0 ± 1.8%). LGG (liver: 5.8 ± 1.0%; spleen: 1.6 ± 0.4%), Bb99 (liver: 13.9 ± 4.3%; spleen: 10.3 ± 3.7%), and EcN (liver: 8.5 ± 3.2%; spleen: 1.0 ± 0.2%) increased the percentage of both the hepatic and splenic T-helper 17 cells. Moreover, LGG (85.5 ± 3.0%) and EcN (89.6 ± 1.2%) increased the percentage of splenic regulatory T-cells. The tested mixtures of the probiotics had different immunological effects from their individual components on cell-mediated responses and cytokine production. In conclusion, our results confirm that the immunomodulatory potential of the probiotics is strain- and organ/tissue-specific, and the effects of probiotic mixtures cannot be predicted based on their single constituents.

## Introduction

Probiotic bacteria are living micro-organisms that confer health benefits on hosts upon consistent and sufficient administration (1–3). Probiotics have been shown to have moderate ameliorating or preventing effects in several diseases, including infectious- and antibiotic-associated diarrhea (4–6), childhood infections (7, 8), enterocolitis (9), inflammatory bowel diseases (10, 11), allergic diseases (12–15), and depression (16). The variety is mind-boggling but in line with the broad impact of gut-associated host-microbe interactions on the immunological, physiological, and anatomical phenotypes of the hosts (17, 18). Many of the observed clinical effects of the probiotics may be due to their immunological effects such as activating inflammation (19–22), antibody production (23, 24), or normalizing polarized helper T-cell responses (25, 26). Yet, the utilization of probiotics as immunomodulatory agents or adjunct immunotherapy is far from its potential (27–29).

One largely neglected area of the research is the immunotherapeutic potential and safety of the probiotics in hepatic conditions. The researchers may have overlooked the plausibility that the probiotics, their metabolites, or antigenic components may have direct access to the liver from the gut *via* the portal vein. Indeed, there is an indication that specific probiotics may affect natural killer (NK) and natural killer T (NKT)-cell populations in the liver (30) but less is known about their influence on the hepatic T-cell subpopulations.

The selection of probiotics for clinical trials is often arbitrary and the same strains are studied, almost as a custom, in multiple diseases even with opposing immunological etiologies. This shows underappreciation of the long-known strain specificity of the immunological effects of the probiotics (31, 32). There is also a shortage in comparative profiling of the immunological effects of different probiotic strains, which limits the possibility for evidence-based selection strains for clinical intervention trials.

A further complication to the selection process is brought by the increasing interest in using mixtures of probiotic strains over single-strain preparation. This interest is perhaps primarily driven by intuition based on the well-documented association of reduced gut microbial diversity with lower colonization resistance and a variety of disease states (17, 33, 34). However, regarding the efficacy of the probiotics which are allogenic microbes, this view is likely to be too simplistic, especially regarding the immunomodulatory properties. We hypothesize that the immunomodulatory effects of a mixture of probiotics are not directly derivable from the effects of its single-strain constituents and for specific immunostimulatory effects. The single strains might have a greater potential than the mixtures. This study aims to address these issues and to present a comparative analysis of the selected probiotics and their mixtures on systemic and liver-immune profiles in healthy C57BL/6 mice on a normal diet.

## Materials and Methods

### Animals

The male C57BL/6 mice aged 6 week old (*n* = 5–11) were assimilated for 1 week before the experiment. They were kept in plastic cages in an air-conditioned room and given free access to food and water. All procedures were performed according to the national legislation on animal experimentation and approved by the Committee of Animal Experiments in the County Administrative Board (the State Provincial Office of Finland, Hämeenlinna, Finland, permit #0703276).

### Probiotic Strains and Feeding Procedures

*Lactobacillus rhamnosus* GG (LGG) (1.7 × 10^12^ colony-forming units (CFU)/g), *Propionibacterium freudenreichii* ssp. *shermanii* JS (PJS) (2.2 × 10^12^ CFU/g), *Lactobacillus rhamnosus* LC705 (LC705) (2 × 10^11^ CFU/g), and *Bifidobacterium breve* 99 (Bb99) (4.2 × 10^11^ CFU/g) were provided by Valio Ltd (Helsinki, Finland) while *Escherichia coli* Nissle 1917 (EcN) (1 × 10^11^ CFU/g) was provided by Ardeypharm GmbH, Germany. These strains were selected as (1) they represent a heterogeneous array of different bacterial genera including the representatives of the most common genera, *Lactobacillus* and *Bifidobacterium*; (2) the gram-negative cell wall of an *Escherichia* strain would be expected to act *via* different receptors than the gram-positive strains; and (3) a *Propionibacterium* strain could have a special immunomodulatory potential due to its very high genomic GC content (>67%) (35, 36). To address the strain specificity, two closely related strains, namely LGG and LC705 were included in the study. A known difference between these two strains is the better mucosal adherence capacity of LGG. We also studied two combinations of these probiotics: a previously studied mixture (37) of LGG, PJS, LC705, and Bb99 (referred to here on as GGmix), and a novel mixture of PJS and EcN (referred to here on as ECPJSmix) comprising the representatives of the less typical probiotic genera.

The suspensions with probiotic bacteria were freshly prepared each day by weighing a defined amount of freeze-dried powders with a known concentration (as confirmed by serial dilution plate culturing) and suspending that in sterile saline. The probiotic mixtures were prepared by mixing equal amounts of the freshly prepared single-probiotic suspensions. The mice were administered with a total dose of 10^7^ CFU of probiotic bacteria in 150–200 μl of saline (6–8 ml/kg) per day by a syringe fitted with an oral gavage needle (75-7202, Scanbur A/S, Karlslunde, Denmark) for 3 consecutive weeks by experienced animal laboratory personnel. Based on a body surface area normalization method, the dose corresponds to approximately 2–3 × 10^9^ CFU of probiotic bacteria per day for an adult human (38). The control group mice were only administered sterile saline.

A total of 71 mice were used in the experiment, of which 11 mice were excluded (*n*_control_: 11; *n*_LGG_: 7; *n*_LC705_: 10; *n*_PJS_: 5; *n*_Bb99_: 5; *n*_EcN_: 6; *n*_GGmix_: 11; *n*_ECPJSmix_: 5) due to visibly poor compliance to the gavaging procedure resulting in visible stress and difficult or impossible dosing, declining health including signs of dehydration and weight loss, and in some cases, esophageal damage (confirmed by autopsy). These dosing-procedure-associated side effects did not differ significantly between the treatment groups and the control groups.

### Hepatocytes and Splenocytes

The mice were sacrificed by cervical dislocation. The liver and splenic lymphocytes were isolated as done previously with minor modifications (31, 32). In brief, livers and spleens were homogenized and washed two times with the RPMI-1640 medium (300 × g for 10 min at 4°C). The OptiPrepTM working solution (OPWS)-40% iodixanol (Sigma Chemical, St. Louis, Mo., USA) was added to the liver pellets and then layered with Hank's balanced salt solution (Sigma) while the red blood cell lysing buffer (Sigma R7757) was added to the spleen pellets. The pellets were then washed at least two times with the RMPI-1640 medium (300 × g for 7 min at 4°C) and stained with anti-mouse antibodies, including CD3-Alexa 700, CD4-APC/Cy7 or CD4-FITC, CD8-PE/Cy7, NK1.1-PerCP/Cy5.5, CD25-PE, IFNγ-Alexa Fluor 488, IL4-PE, IL17-Alexa Fluor 647, and FOXP3-APC (all from eBioscience). The staining procedures and analyses were performed according to the manufacturer's protocol. The samples were analyzed by the FACSCalibur flow cytometer (BD Biosciences) as previously described (39).

### Blood Cytokines

The blood samples for the serum analyses were collected from the submandibular vein by the Microvette CB 300 capillary tubes according to the manufacturer's instructions (Sartsedt AG & Co, Nümbrecht, Germany) before the mice were sacrificed. The serum levels of 22 cytokines and chemokines were measured using the Mouse Cytokine/Chemokine Premixed Linco_*plex*_ kit (Cat.# MCYTO-70K-PMX; Millipore Billerica, MA) with standard protocol as previously described (40).

### Statistical Analyses

Data were presented as mean ± SEM. Kruskal–Wallis test and Mann–Whitney test were used to evaluate the significant differences between the probiotic treatment groups in reference to the control groups. Statistical significance of the observed differences was evaluated at 10% (^*^) and 5% (^**^) significance levels without adjustment for multiple comparisons and at a 5% significance level with Bonferroni adjustment (^***^). Statistical calculations were performed using SPSS 20 for Windows (Chicago, IL, United States) and GraphPad Software Prism 5.04 (San Diego, CA, United States).

## Results

### Effects on the Body, Spleen, and Liver Weights

**Figure 1:**
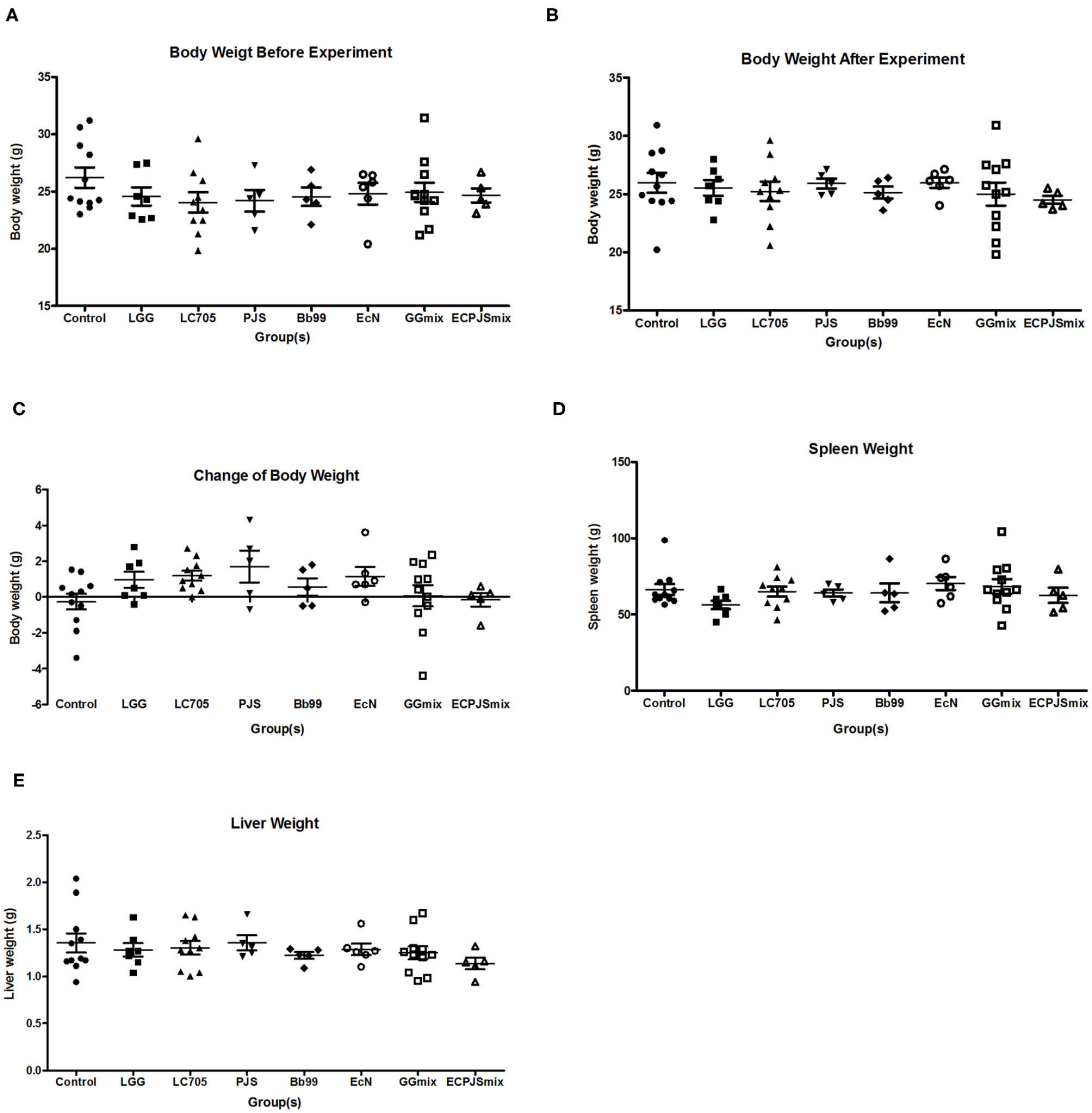
Weight analysis of the body, spleen, and liver of the mice. **(A)** Body weight of the mice before the experiment, **(B)** body weight of the mice after the experiment, **(C)** change of body weight of the mice, **(D)** spleen weight of the mice, and **(E)** liver weight of the mice were shown (mean ± SEM, *n*_control_: 11; *n*_LGG_: 7; *n*_LC705_: 10; *n*_PJS_: 5; *n*_Bb99_: 5; *n*_EcN_: 6; *n*_GGmix_: 11; *n*_ECPJSmix_: 5). The statistical significance of the observed differences was evaluated and *** indicates the Bonferroni corrected *p*-value (α = 0.05).

None of the treatments showed significant effects on weight gain during the treatment or the spleen and liver weights after the treatment as compared to the control group (Figure 1).

### Effects on Hepatic and Splenic NK, NKT, and CD8+ Cells

**Figure 2:**
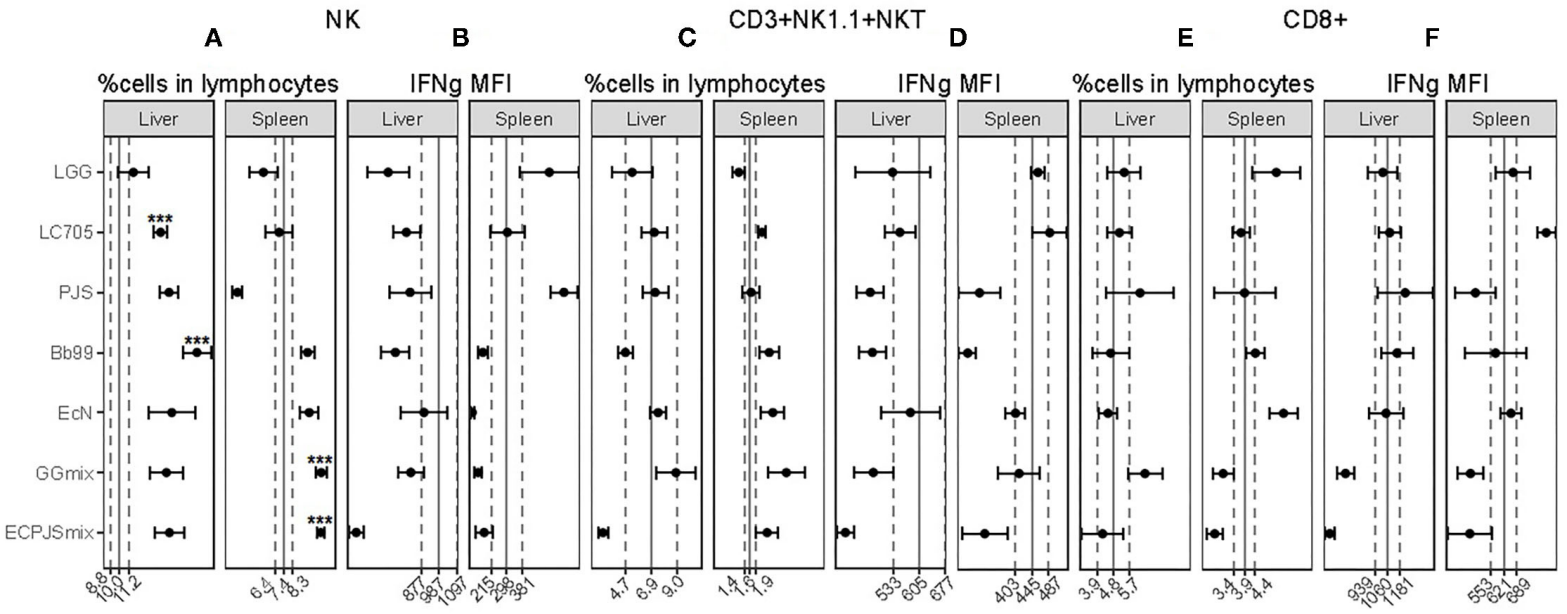
The effects of the probiotics on the NK, CD3+NK1.1+NKT, and CD8+ cell populations. The percentages of **(A)** NK, **(C)** CD3+NK1.1+NKT, and **(E)** CD8+ cells in the lymphocyte population and IFNg mean fluorescence intensity (MFI) within **(B)** NK, **(D)** CD3+NK1.1+NKT, and **(F)** CD8+ cells in the liver and spleen were shown (mean ± SEM, *n*_control_: 11; *n*_LGG_: 7; *n*_LC705_: 10; *n*_PJS_: 5; *n*_Bb99_: 5; *n*_EcN_: 6; *n*_GGmix_: 11; *n*_ECPJSmix_: 5). The vertical solid lines indicate the mean value of the respective control groups. The vertical dotted lines indicate the SEM of the respective control groups. Statistical significance of the observed differences was evaluated and *** indicates the Bonferroni corrected *p*-value (α = 0.05).

As shown in Figure 2A, the percentage of NK cells in both the hepatic and splenic lymphocyte populations was higher in the GGmix-treated group than those in the control group. In the ECPJSmix group, the percentage of NK cells was increased in the splenic lymphocyte population only. For the individual strains, the percentages of NK cells in LC705-, Bb99-, and EcN-treated groups were higher than those in the control group in the hepatic lymphocyte population, with the Bb99-treated group showing the most prominent effect. The percentage of NK cells in the LGG-treated group was comparable to the control group. The percentage of NK cells in the PJS-treated group was increased in the hepatic lymphocyte population but decreased in the splenic lymphocyte population. IFN-gamma (IFNg) production, as indicated by mean fluorescence intensity (MFI), of the hepatic NK cells in the ECPJSmix-treated group decreased whereas that of the splenic NK cells in the PJS-treated group increased (Figure 2B).

The percentage of CD3+NK1.1+NKT cells in the ECPJSmix-treated group was significantly lower than that in the control group in the hepatic lymphocyte population whereas in the LC705- and GGmix-treated groups, the percentage of these cells was increased in the splenic lymphocyte population (Figure 2C). IFNg MFIs of the splenic CD3+NK1.1+NKT cells in the PJS-, Bb99-, and ECPJSmix-treated groups were depressed in comparison with the control group (Figure 2D).

No significant differences were observed in the percentage of the CD8+ cells or the IFNg MFIs of the CD8+ cells in the hepatic lymphocyte population between any of the probiotic-treated groups and the control group. Among the splenic lymphocytes, the percentage of the CD8+ cells was increased in the EcN-treated group (Figure 2E). The IFNg MFI of the splenic CD8+ cells was increased in the LC705-treated group and decreased in the GGmix-treated group in comparison with the control group (Figure 2F).

### Effects on the Helper T (TH)-Cells

**Figure 3:**
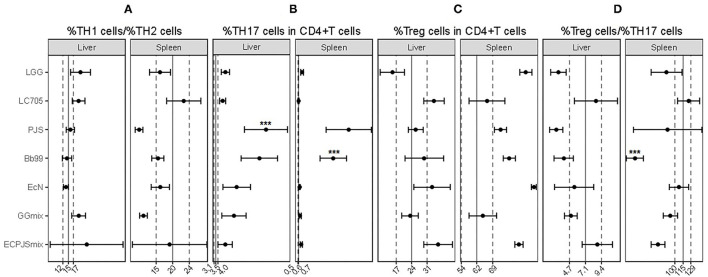
The effects of probiotic treatments on helper T-cell populations. **(A)** The ratio of %TH1 cells to %TH2 cells; percentages of **(B)** TH17, and **(C)** regulatory T (Treg)- cells in all CD4+ T-cells; and **(D)** the ratio of %Treg cells to %TH17 cells in the liver and spleen were shown (mean ± SEM, *n*_control_: 11; *n*_LGG_: 7; *n*_LC705_: 10; *n*_PJS_: 5; *n*_Bb99_: 5; *n*_EcN_: 6; *n*_GGmix_: 11; *n*_ECPJSmix_: 5). The vertical solid lines indicate the mean value of the respective control groups. The vertical dotted lines indicate the SEM of the respective control groups. Statistical significance of the observed differences was evaluated and *** indicates the Bonferroni corrected *p*-value (α = 0.05).

It seemed that none of the probiotic treatments influenced the TH1/TH2 balance in the liver or spleen (Figure 3A). In contrast, all the treatment groups, except LC705, showed an increase in the percentage of TH17 cells in either or both tested organs when compared with the control group (Figure 3B). Moreover, the percentages of the splenic regulatory T (Treg)-cells in the LGG-treated and EcN-treated groups were higher than those in the control group (Figure 3C). Nonetheless, the splenic Treg/TH17 ratio of the LGG-, Bb99-, and ECPJSmix-treated groups was lower than that in the control group (Figure 3D).

### Effects on Serum Cytokine and Chemokine Levels

**Figure 4:**
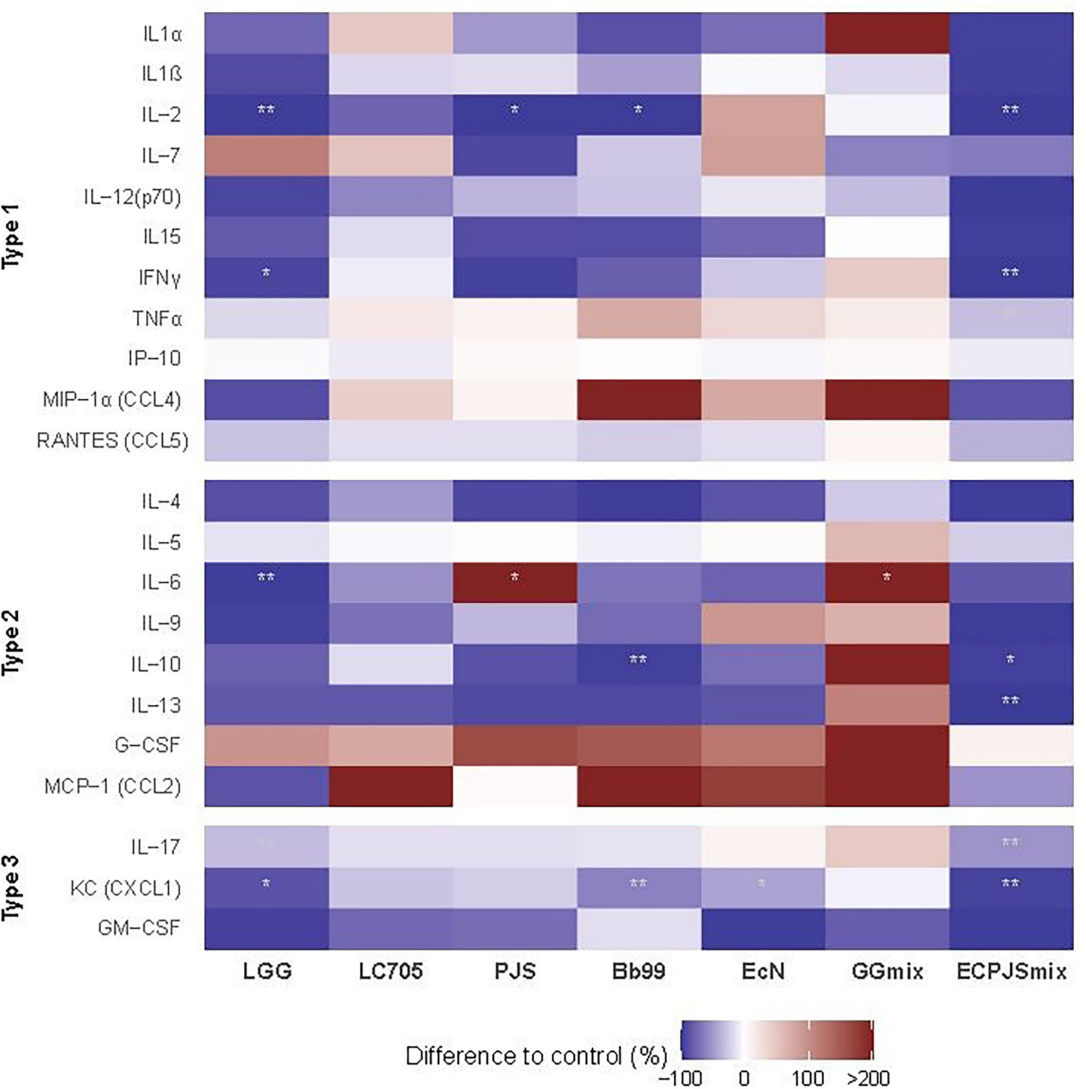
The effects of the probiotics on the serum cytokine and chemokine levels. The cytokine levels presented as percent difference in the geometric mean of the cytokine levels compared to controls (control = 0) in a heat map (n_control_: 11; *n*_LGG_: 7; *n*_LC705_: 10; *n*_PJS_: 5; *n*_Bb99_: 5; *n*_EcN_: 6; *n*_GGmix_: 11; *n*_ECPJSmix_: 5) with red indicating higher and blue indicating lower mean levels compared to the control. One observation of IL-10 in the GGmix group with mean > + 2SD was excluded. For a rough indication of the nature of the studied cytokines, they are divided into type 1 cytokines, referring to primary involvement in the innate and cell-mediated and TH1-associated immune responses, type 2 cytokines referring to involvement in the humoral and TH2-associated immune responses, and type 3 immunity referring to TH17-mediated immune responses or more than one type of immune response. Statistical significance of the observed differences was evaluated at 10% (*) and 5% (**) significance levels without adjustment for multiple comparisons and at s 5% significance level with Bonferroni adjustment (***). CCL, chemokine C-C motif ligand; CXCL, C-X-C motif chemokine ligand; G-CSF, granulocyte colony-stimulating factor; GM-CSF, granulocyte-macrophage colony-stimulating factor; IFN, interferon; IL, interleukin; IP, interferon-inducible protein; KC, keratinocyte-derived chemokine; MCP, monocyte chemoattractant protein; MIP, macrophage inflammatory protein; RANTES, regulated upon activation, normal T-cell expressed and secreted; TNF, tumor necrosis factor.

The effects of the probiotics on the serum cytokine and chemokine levels are shown in Figure 4. In comparison with the control, lower serum levels of IL-2 were seen in the LGG-, ECPJSmix-, Bb99-, and PJS-treated groups; KC in the LGG-, Bb99-, EcN-, and ECPJSmix-treated groups; IFNγ and IL-17 in the LGG- and ECPJSmix-treated groups; IL-10 in the Bb99- and ECPJSmix-treated groups; IL-6 in the LGG-treated group; and TNFα and IL-13 in the ECPJSmix-treated group. Only the level of IL-6 in the PJS- and GGmix-treated groups was higher than that in the control group.

## Discussion

This study provided several key observations. First, the study confirms that probiotics have the potential to affect the relative abundance and activity of lymphocyte populations in the liver and spleen. Second, this potential varies between different probiotic species and even between closely related strains (LGG vs. LC705). Third, probiotic supplementation may concomitantly increase the pool of a particular lymphocyte population in one organ while decreasing it in another organ. Fourth, the probiotics appeared to increase the relative abundance of the hepatic NK cells and TH17 cells in the liver and spleen in a nearly universal manner. Fifth, LGG and EcN increased the relative abundance of the Treg cells in the spleen. Sixth, the studied probiotics had little influence on the TH1/TH2 balance in this animal model. Seventh, the effects of the two mixtures studied were different from their individual components but tended to be in the same direction with each other.

In the study, LC705, PJS, Bb99, EcN, and GGmix increased the percentage of the NK cells in the liver with Bb99 showing the most prominent effect. The potential of specific probiotics to stimulate the NK cells that are known central anti-viral and anti-tumor cells of the innate immune system is in line with some previous studies. For example, *B. adolescentis* BBMN23 and *B. longum* BBMN68 have been shown to increase the NK cell activity in the BALB/c mice (41) and *B. longum* SP 07/3 has been shown to enhance the NK cell activation and activity in the *ex vivo* models with human peripheral blood mononuclear cells (PBMCs) (42). *Lactobacillus casei* Shirota (LcS) have been shown to increase the NK cell activity in the peripheral blood of smokers and patients with biliary cancer surgery (43–45) and to increase the cytotoxicity of the NK cells in the mouse spleen (46). However, in healthy adults, LcS have been shown to depress the lytic activity of the NK cells (44). LGG had little influence on the splenic or hepatic NK cells in our study, while it has been shown to increase the activity of the NK cells in the *in vitro* model with human PBMCs (47).

LGG, Bb99, and EcN were all shown to increase the percentage of the TH17 cells in both the liver and spleen but the PJS increased the percentage of the TH17 cells in the liver only. The cytokine measurements indicated, however, that in the peripheral blood, the TH17 cell-signature cytokine was decreased by LGG treatment, possibly indicating a lower peripheral activity or drainage of the peripheral pool of the TH17 cell to the other tissues such as the liver and spleen as indicated by the cellular data. LGG has been previously shown to increase the percentage of the TH17 cells when co-cultured with human PBMCs (48). Also, a higher percentage of the TH17 cells was observed in the spleens of LGG groups when compared to that of the control groups in Shi et al.'s study (49). In contrast, the probiotic strains, *Enterococcus faecalis* FK-23 and *Bifidobacterium infantis*, have been associated with a reduced TH17-immune-response in different animal models (50, 51). The TH17 cells play an important role in defense against extracellular pathogens (52), and in this regard, their activation in response to probiotic administration is logical. While an increase in the abundance of the TH17 cells could improve host defense against bacterial infection, the chronic effects of long-term consumption of the probiotics should be further investigated in clinical trials as an inappropriate or uncontrolled activation of the TH17 response is linked to proinflammatory and autoimmune diseases (53–56).

Notably, LGG and EcN increased the percentage of the Treg cells in the spleen. Tregs are known as the key immunoregulatory cells in suppressing immune responses and maintaining immunological homeostasis. This is in line with some previous studies. For example, the LGG administration has been associated with increased Treg activity in the murine model with asthma (57) and ovalbumin-immunized rats (58) and EcN treatment with an increase in the intradermal Treg cells in the mouse model of atopic dermatitis (59). Also, in the study of Shi et al., the mice in the low-dose LGG group had a higher percentage of Treg cells in the spleens than those in the control group and high-dose LGG group (49). Previously another *Bifidobacterium breve* strain has been shown to prevent the decrease in the splenic and intestinal Tregs associated with food allergen sensitization in the mouse model of food allergy without a significant effect in the naïve mice (60). In our study, the Bb99 treatment was associated with a non-significant increase in the percentage of the Treg cells and significantly reduced Treg/TH17 cell ratio in the spleen, indicative of an anti-inflammatory potential for the studied strain.

Only a few associations were observed in our study between the different probiotic treatments and other studied effector cells, namely CD8+ cells and CD3+NK1.1+NKT cells. As cytotoxic cells, CD8+ cells act especially against intracellular pathogens. Thus, it would seem rational that exposure to non-invasive probiotic bacteria would primarily not activate them. NKT cells are important players in the first line of innate defense against bacterial and viral infections and bridge the innate and adaptive immune system. Their activation by probiotics has been suggested to improve high-fat diet-induced hepatic steatosis and insulin resistance (30).

We also investigated the effects of probiotics in cytokine profiles. Interestingly, the closely related *Lactobacillus rhamnosus* strains, that is, LGG and LC705, had distinctly different effects. The more adherent LGG strain was associated with nearly universal suppression of all cytokine levels while LC705 had less effect. It was also interesting that some of the components of the GGmix particularly, LGG and Bb99, affected some of the cytokine levels, for example, both LGG and Bb99 reduced the level of neutrophil chemoattractant KC, but the mixture did not seem to affect any cytokine levels. This could be partly because of the lower dose of the individual strains in the mixture. However, it could also indicate that exposure to a single bacterial strain elicits a different response than exposure to the bacterial mixture and thus a concomitant encounter of a wider array of different immunogenic signals. Previously, a combination of LGG and LC705 has been shown to be more immunogenic to human monocyte-derived macrophages than the individual strains (61). ECPJSmix was associated with a prominent universal decrease in the cytokine levels compared to the control and as with GGmix, the effect of the mixture was distinct from that of its two individual components. Further studies are called for to investigate the differences in the immunological effects following exposure to individual bacterial strains or their mixture. Overall, there is a shortage of comparable studies on the effects of the probiotics studied here on the peripheral cytokine profile in healthy mice. However, the tendency of the probiotics, LGG, and the ECPJSmix, in particular, to reduce the proinflammatory cytokine levels is in agreement with the potential anti-inflammatory effects associated with LGG and many other probiotics in clinical studies (62–64). *In vitro* probiotics tend to stimulate the secretion of a wide range of cytokines from the cultured PBMCs, including the proinflammatory cytokines (25). Interestingly in one comparative study of different probiotics, including EcN and LGG, the LGG-derived extracts and cell debris were shown as the weakest inducers of proinflammatory cytokines (65), which is in line with our results.

All the studied individual strains and GGmix have a long history of safe human use and the ECPJSmix combination created for experimental purposes is the only exception. For this reason, a safety assessment of these strains was out of the scope of this study. None of the treatments were seen to influence weight gain or the weight of the studied organs: spleen and liver. However, gavaging *per se* was associated with visible stress in some of the mice and the effect of this on the results is unknown. The stress would be expected to be equal between the treatment groups and all mice that responded poorly to the procedure were excluded. The number of excluded mice did not significantly differ between the treatment groups and the control group. Nonetheless, while oral gavaging was chosen here to guarantee comparable dosing of the probiotics in each mouse, we strongly recommend against using it in similar experiments in the future where the effects of daily administration of food supplements are addressed.

In conclusion, this study confirms that the immunomodulatory potential of probiotic bacteria is strain- and organ/tissue-specific. Therefore, the immunomodulatory properties of the probiotic candidates should not be extrapolated from the observations obtained with the other strains or even with the same strain in different target organ/tissue. Based on the animal model and the probiotic strains and their combinations in our study, probiotic supplementation would seem to have the most potential to influence NK, TH17 cell-, and Treg cell-mediated immune responses both systemically and in the liver, but little influence on the TH1/TH2 balance. The influence on the phagocytosing cells was not studied here and it should be addressed in future studies. Our study indicated that the immunological effects of the probiotic mixtures can be dramatically different from that of their individual components; and thus, the effects of a probiotic mixture cannot be extrapolated from those of its individual strains. The specific immunological effects may be different with human exposure and different dosages than what was observed here in the mice with a single dose. Therefore, the presented specific immunological effects should be only utilized as supporting evidence and for hypothesis generation. However, for general guidance, our results highlight that the immunomodulation by the probiotic bacteria is strain-specific as well as organ/tissue-specific and that the effects of the probiotic mixtures cannot be predicted based on the effects of their single constituents.

## Data Availability Statement

The raw data supporting the conclusions of this article will be made available by the authors, without undue reservation.

## Ethics Statement

The animal study was reviewed and approved by Committee of Animal Experiments in the County Administrative Board (The State Provincial Office of Finland, Hämeenlinna, Finland).

## Author Contributions

PK designed the study with contributions from HE-N. PK supervised and OM contributed to the laboratory work. Result analyses were primarily carried out by FF with contributions from OM, HE-N, and PK. The manuscript was written by FF and PK with important contributions to the intellectual content from OM and HE-N. All authors contributed to the article and approved the submitted version.

## Funding

This study was supported by grants from the Academy of Finland (Number 122015), the Finnish Cultural Foundation (Yrjö Similä Fund), Emil Aaltonen Foundation, and the General Research Fund (GRF) from the Research Grant Council (GRC) of Hong Kong (Number 765810).

## Conflict of Interest

The authors declare that the research was conducted in the absence of any commercial or financial relationships that could be construed as a potential conflict of interest.

## Publisher's Note

All claims expressed in this article are solely those of the authors and do not necessarily represent those of their affiliated organizations, or those of the publisher, the editors and the reviewers. Any product that may be evaluated in this article, or claim that may be made by its manufacturer, is not guaranteed or endorsed by the publisher.
